# A Finite Element Study Of Transient Wave Propagation in Plates

**DOI:** 10.6028/jres.092.025

**Published:** 1987-08-01

**Authors:** Mary Sansalone, Nicholas J. Carino, Nelson N. Hsu

**Affiliations:** National Bureau of Standards, Gaithersburg, MD 20899

**Keywords:** finite element analysis, Green’s function, impact, impact-echo method, plate response, stress wave propagation

## Abstract

Studies of transient wave propagation in plates were carried out to establish a basis for the impact-echo technique as a nondestructive test for flaw detection in concrete. The surface displacements caused by stress waves generated by point impact on a plate were calculated using both the Green’s function solution and the finite element method; displacement waveforms obtained by the two approaches showed good agreement. Displacement and stress fields in a plate were studied using finite element analysis. It was shown that transient point load applied normal to a stress-free boundary gives rise to P- and S- “wakes”—disturbances trailing the P- and S-waves. The displacement and stress fields in each wake resemble those in the preceding wave.

## Introduction

For more than 30 years, efforts have been made to apply stress wave propagation to nondestructive testing of concrete. These efforts have met with limited success, although some progress has been made in measuring the thickness of plate elements and for integrity testing of rod-like structures, such as piles [1]^1^. Progress has been limited because of the heterogeneous nature of concrete, which strongly attenuates high frequency waves; thus traditional wave propagation methods developed for flaw detection in metals cannot be used for evaluation of concrete.

The National Bureau of Standards has been working to develop a nondestructive test method for concrete using transient stress waves [2–4]. This method is referred to as the impact-echo method. The technique involves introducing a transient stress pulse into a test object by mechanical impact at a point and monitoring reflections of the pulse from internal defects and external boundaries. Stress pulses with sufficient energy have been generated by dropping small diameter (4–16 mm) steel spheres (ball bearings) onto concrete.

The impact-echo test is a simple procedure; however, successful interpretation of displacement waveforms requires an understanding of the interaction of transient stress waves with internal defects. The current state of knowledge about the propagation of transient stress waves in bounded solids containing defects is very limited. Thus the NBS program has focused on understanding the nature of transient stress wave propagation in solids containing defects as well as on the implementation of the impact-echo method. In a current phase of the program, the finite element method is being used to study displacement and stress fields generated by point impact on an elastic solid and the interaction of transient waves with internal discontinuities and stress-free boundaries.

This paper presents results of finite element analysis of transient stress wave propagation in plates. To verify the analyses, surface displacement time histories obtained from the finite element method are compared to exact Green’s function solutions for impact on an infinite plate. A second paper, also appearing in this issue of the *Journal of Research*, presents a finite element study of the diffraction of transient waves by planar flaws in a plate.

## Background

### Transient Wave Propagation

**Figure 1:**
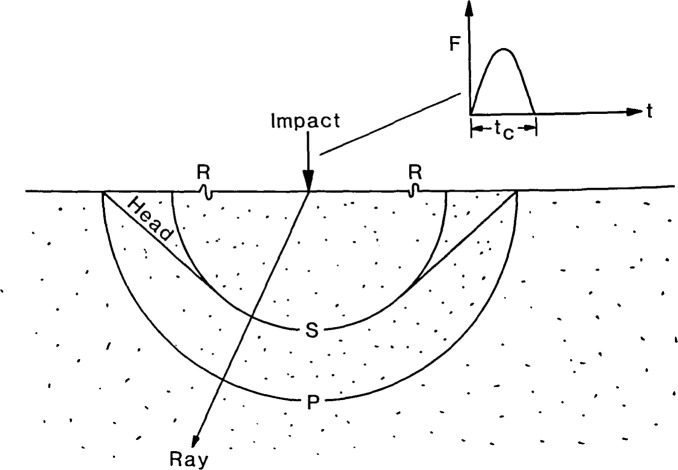
Schematic representation of the wavefronts produced by point impact on a semi-infinite solid.

Point impact on the surface of a solid gives rise to three types of transient disturbances: dilatational and distortional waves which propagate into the solid along spherical fronts, and a Rayleigh (R) wave which propagates along a circular front over the surface of the solid. The dilatational and distortional waves are commonly referred to as P- and S-waves. In addition, there is a low amplitude wave known as a head wave. The front of the head wave extends from the intersection of the P-wavefront with the surface of the solid to a point that is tangent to the S-wavefront. Figure 1 is a schematic representation of the P-, S-, R- and head wavefronts generated by a point impact on an elastic solid.

P- and S-waves are characterized by the direction of particle motion with respect to the direction the wavefront is propagating. In the P-wave, displacement is parallel to the direction of propagation; in the S-wave, the motion is perpendicular to the direction of propagation. These waves travel at different speeds; their relative speeds depend on the Poisson’s ratio of the material being tested. For a Poisson’s ratio of 0.2, which is a typical value for concrete, the S- and R-waves travel at approximately 61% and 56% of the P-wave speed, respectively [1].

The P- and S-waves are reflected by stress-free boundaries and by internal defects of sufficient size. For example, in a plate, multiple reflections occur as the waves travel back and forth between the two free surfaces. This type of reflection is referred to as specular reflection. When a P-wave strikes a boundary at an oblique angle, an S-wave can also be produced by the process of mode-conversion. Likewise, an incident S-wave produces a P-wave. The angles of specularly reflected and mode-converted waves are determined by Snell’s Law [5]. A receiving transducer located on the top surface of the plate, near the point of impact, responds to the surface displacements caused by the successive arrivals of each reflection from the bottom of the plate.

**Figure 2:**
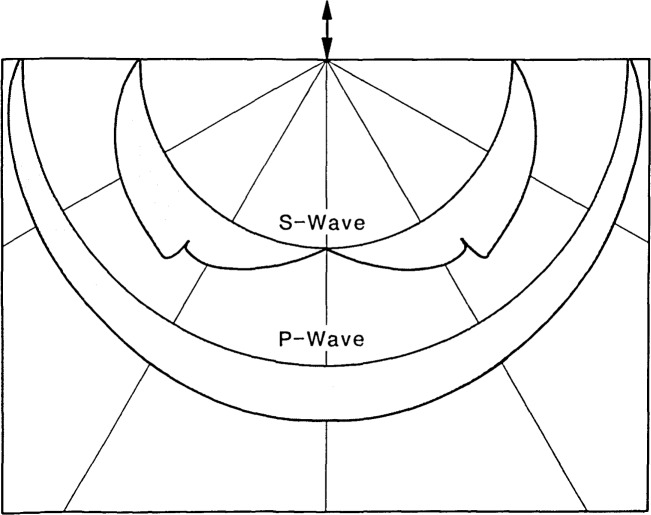
Amplitude of particle displacements in the radiation pattern produced by a harmonic point source.

Due to the complexity of the problem, explicit equations for the radiation pattern produced by a transient point source on a semi-infinite solid have not yet been derived. Ideas about the nature of this radiation pattern come from knowledge about a harmonic point source on a semi-infinite solid [6–8]. Figure 2 shows the angular variation of the amplitude of displacements within the P- and S- waves for a material with Poisson’s ratio equal to 0.2. In the P-wave, the amplitude of the displacements is maximum at the centerline of the plate and decreases to zero at the surface. In the S-wave, the amplitude of displacements is zero at the centerline of the plate and at the surface and is maximum along a ray located approximately 40 degrees from the centerline. There is a discontinuity in the S- wave displacements at an angle, *θ_c_*, given by the following equation:

$$\theta_{c}=\arcsin\;(C_{s}/C_{p})$$ (1)

where

*C*_s_: =S-wave speed, m/s; and
*C*_p_: =P-wave speed, m/s.

In this paper, the finite element method is used to study the nature of the internal displacement and stress fields produced by a transient point load on a plate.

### Green’s Function Solutions

Theoretical solutions for transient wave propagation in solids are available for a limited number of problems; these solutions can be used to obtain the displacement response at points in a solid. The displacement, *u*(*r,t*), at a point due to an impact at some other point on an elastic body can be represented by a convolution integral:

$$u(r,t)=\int_{0}^{t}G(r,t-\tau )F(\tau )d\tau$$ (2)

where *F*(*t*) is the impact force as a function of time and G (*r,t*) is the dynamic Green’s function of the elastic body. The Green’s function is defined as the impulse (dirac delta function) response of the body for a particular impact configuration (impact at one location and the receiver at a different location).

The Green’s function solution is the exact solution to the partial differential equations and associated boundary conditions governing elastic wave propagation. Green’s function solutions can be obtained using Generalized Ray Theory^2^. The solution is in the form of an infinite series expansion. Stress waves can be visualized as propagating along ray paths. Each term in the series corresponds to the arrival of successive stress waves which propagate along the various ray paths that connect the impact source to the receiver. For a given time duration, a finite number of rays (terms in the series expansion) contribute to the total displacement response at the receiver.

Explicit formulae for Green’s function solutions which are amenable to numerical computations have been derived only for simple geometries, such as a semi-infinite space or an infinite plate. (For computation of the Green’s function, see refs. [10,11]. To obtain displacement and stress fields in bounded solids, the finite element method was used.

### Finite Element Method

The finite element method is a general numerical technique for obtaining approximate solutions to the partial differential equations that arise from boundary value problems. The method involves dividing a continuum into a finite number of discrete parts—the finite elements. The discretized representation of the continuum is referred to as the finite element model. For stress analysis, the behavior of each element is described by a set of assumed functions which represent the variation of displacements or stresses within that element. Variational (or energy) principles are used to formulate forcedisplacement element equations. These element equations are then used to construct the global equations which describe the behavior of the entire continuum. Solution of these global equations gives the displacements or stresses at points in the element [12].

An explicit, two-dimensional (axisymmetric or plane strain), finite element code (DYNA2D), developed at Lawrence Livermore Laboratory for solving finite-deformation, dynamic contact-impact problems [13–15], was used to perform the studies discussed in this paper. An input generator (MAZE) [16] was used to create the finite element model. A mini-computer with a virtual operating system, 8 MBytes of memory, and a floating point processor were used to carry out the analyses.

IN DYNA2D, a continuum is divided into elements using constant strain (linear displacement) triangles and quadrilaterals [13]. Higher order elements (e.g., linear strain, quadratic strain) are not available in DYNA2D because they are computationally expensive in wave propagation applications relative to the constant strain elements. For a particular element type, the accuracy of the finite element solution is partly determined by element size. In wave propagation problems, the optimum element size depends on the geometry of the continuum and on the time-history of the dynamic loading. For the constant strain quadrilaterals and the dynamic loading functions used in the linear elastic, plate analyses presented in this paper, convergence studies were carried out to determine the optimum element size. The criteria for convergence were comparisons made between finite element displacement time-histories obtained at points on the top and bottom surfaces of a plate and the waveforms obtained at the same points by the Green’s function solution for an infinite plate. For 0.25 m to 0.5 m thick plates subjected to a force-time function which simulated impact by a steel sphere (contact time of impact was 25 to 31 *μ*s), rectangular elements with dimensions on the order of 0.02 times the plate thickness were found to give sufficiently accurate results. The elastic material properties used in these analyses were representative of concrete.

In dynamic finite element analyses, numerical integration of the equations of motion must be carried out; DYNA2D uses the central difference method [13] to perform this integration. The central difference method requires a small time step for numerical stability. This is not a drawback because wave propagation applications require the use of very small time steps to obtain an accurate solution. Numerical stability requires that the time step, *h*, meet the following criterion:

$$h\;⩽h_{\max}=L/C_{p}$$ (3)

where

L: = shortest dimension of the element, m; and
*C*_p_: = P-wave speed in the material, m/s.

In DYNA2D, the time step is taken as 0.67/*h*_max_ unless the user specifies some other value. During an analysis, data are stored in data files at intervals specified by the user. In the analyses discussed in this paper, data were stored every 2 *μ*s. An interactive graphic post-processor (ORION) [17] was used to process the results of the analyses.

Before the finite element code could be used with confidence to study transient wave propagation in bounded solids containing internal flaws, solutions obtained from the finite element analyses were verified using the Green’s function solutions for infinite plates.

## Plate Response

The successful implementation of the impactecho technique as a method for flaw detection in heterogeneous materials, such as concrete, requires an understanding of the reflection of transient stress waves by the free boundaries of a solid and the interaction of waves with internal defects. A first step is understanding the response of an infinite, homogeneous plate to impact. In the following discussion of the elastic response of a plate to point impact on the top surface, the following analytical results are presented: 1) the displacement time-history obtained at the bottom surface of the plate directly under the impact point; 2) displacement fields recorded at successive times to show transient stress waves propagating within the plate; and 3) the displacement time-history of a point on the top surface of the plate near the point of impact.

**Figure 3:**
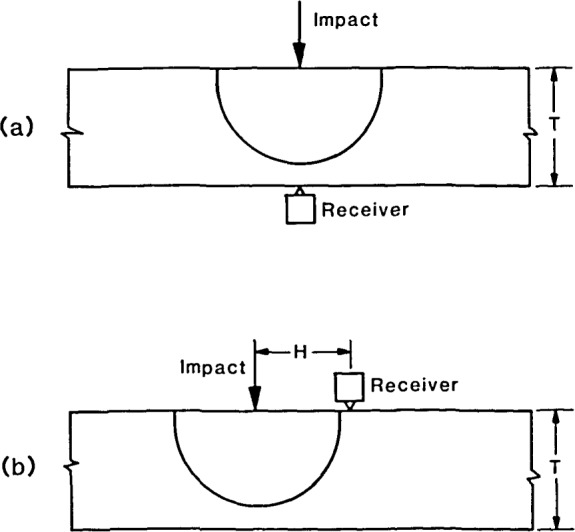
Test configurations for a plate: (a) epicenter; and (b) impact-echo.

For the case of a sphere impacting on a plate, eq (2) can be used to predict the surface displacement that will be detected by a receiving transducer located on either the top or bottom surface of the plate. Two test configurations are considered in this study; these are shown in figure 3. Figure 3(a) shows the receiver located at the epicenter, that is, on the bottom surface of the plate directly under the point of impact. Figure 3(b) shows the impactecho configuration—the receiver is located on the top surface of the plate near the point of impact. For this configuration, the separation between impact point and receiver is denoted by an *H*.

The time-history of the contact force generated by the elastic impact of a sphere dropped on the surface of a plate can be approximated by a half- cycle sine curve (see fig. 1). The contact time of the impact and the maximum contact force can be computed if the size and elastic properties of the sphere, the velocity of the sphere at impact, and the elastic properties of the plate are known [18]. If the appropriate Green’s function, G(*r,t*), is also known, then the displacement, *u*(*r,t*), can be computed by numerical solution of the convolution integral given by eq (2) [19].

In this study, the Green’s function for an infinite plate was obtained using a computer code recently developed at NBS [20,21]. This program computes the response for a unit step force-time function input. To obtain the Green’s function (impulse response), the derivative with respect to time of the step function solution is calculated. The step function response is calculated using nondimensionalized variables so that the solutions are applicable to a plate of any thickness. Values of the step function response are calculated at prescribed time steps so that the computer solution is a discretized representation of the true solution. The only input parameters required are the source-receiver geometry and the ratio of S- to P-wave speeds. In the analyses presented in this paper, the ratio was 0.61 (Poisson’s ratio equal to 0.2).

### Epicenter Response

#### Green’s Function Solution

Before considering the response of a plate to impact by a sphere, the impulse response is shown. In the impulse response, wave arrivals correspond to abrupt discontinuities in the waveform. It is therefore easier to determine the displacements caused by each individual wave arrival.

Since the numerical solution used in this study results in a discrete representation of the step function response, the derivative of this solution (the impulse response) also has a discrete representation.

**Figure 4:**
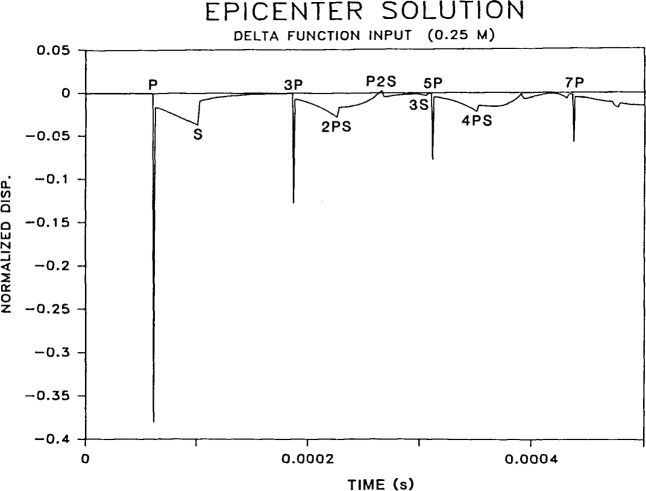
Epicenter response to a delta function impact.

The impulse response for a 0.25 m thick plate is shown in figure 4. The P- and S-wave speeds are 4000 and 2440 m/s, respectively. A time step of 1 *μ*s was used in the calculations. This response consists of normal surface displacements caused by the arrival of direct P- and S-waves, multiply reflected waves (3P, 3S, 5P, etc.) and mode-converted waves (2PS, P2S, etc.). The arrival times of these waves are indicated on the waveform.

The P-wave generated by impact on the top surface of the plate is the first wave to arrive at the epicenter; it is a compression wave (a wave causing compressive stress at the wavefront) and it causes a large downward displacement of the surface. This compression wave will be reflected at the bottom surface of the plate as a tension wave. The tension wave will propagate back up through the plate to be reflected at the top surface as a compression wave. (The multiply-reflected P-wave is now called the 3P-wave because when it arrives at the bottom of the plate it will have traveled through the thickness of the plate three times.) When the 3P-wave arrives at the bottom surface it pushes the surface downward. This cycle is repeated so that every multiply-reflected P-wave arriving at the bottom surface of the plate (5P, 7P, etc.) is a compression wave that causes a downward displacement of the plate surface.

Notice that the amplitude of the surface displacements caused by successive P-wave arrivals decreases. This is due to divergence (spherical beam spreading) which causes the amplitude of the displacement to decrease as the inverse of the distance the wave has traveled [22].

Theoretically, an S-wave arriving at the epicenter has no vertical displacement component (see fig. 2). However, the arrival of the S-wavefront is still easy to identify because the arrival of the wavefront corresponds to a discontinuity in the vertical displacement at the epicenter.

The waveform obtained from the Green’s function solution for a point located a distance *r* from an impulse point source in an infinite solid shows displacements corresponding to the arrival of the P- and S-wavefronts. No other displacements occur. However, in the impulse response of the infinite plate (fig. 4), notice that in addition to the displacements caused by P-, S-, and mode-converted waves, there are displacements that occur between the arrivals of each of these waves. These intermediate displacements are referred to in this paper as “wakes”; they result from the transient point source being applied normal to a stress-free boundary and from the interaction of propagating waves with the lower stress-free boundary of the plate. In the frequency domain these wakes are commonly thought of as geometric dispersion phenomena.

To obtain the epicenter response caused by a sphere impacting the top surface of the plate, the waveform shown in figure 4 must be convolved with the force-time function shown in figure 1. Using the identity for the derivative of convolution, a mathematically equivalent approach, which is numerically more accurate in this case, is to convolve the response function, *H*(*r,t*), computed for a unit step function with the derivative of the force-time function, *dF*(*t*)/*dt* [19]. Thus, eq (2) can be written in the following form:

$$u(r,t)=\int_{0}^{t}H(r,t-\tau )\frac{dF(\tau )}{dt}d\tau$$ (4)

**Figure 5:**
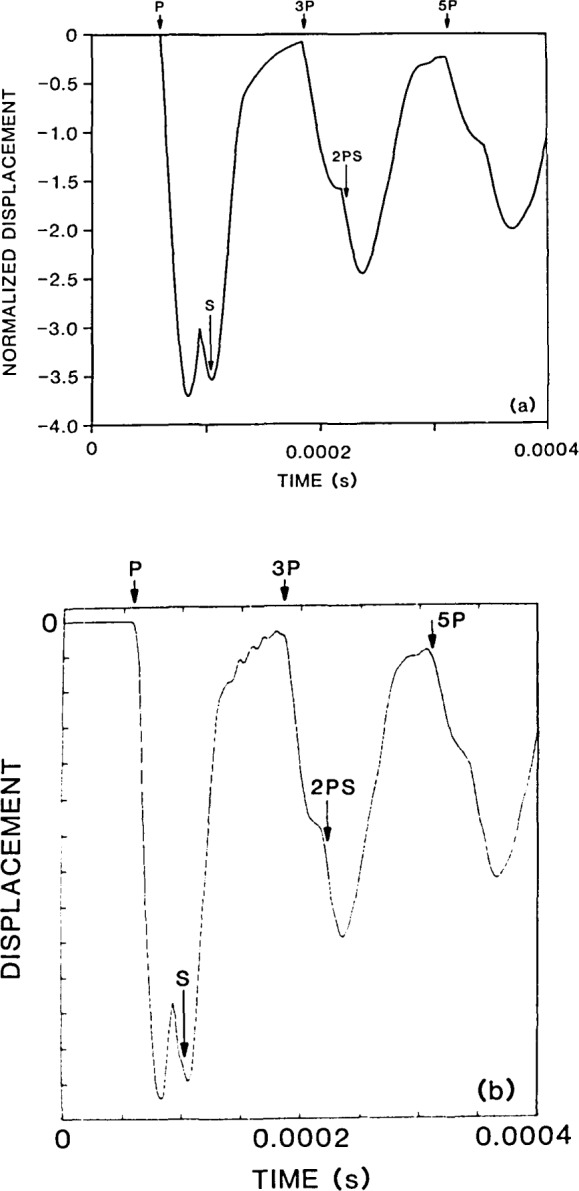
Epicenter response to impact: (a) Green’s function solution; and (b) waveform obtained from finite element analysis.

In this case, the derivative of the force-time function is a half-cycle cosine curve. The waveform obtained by this convolution is shown in figure 5(a). The time step used in these calculations was 2 *μ*s.

In this analysis, the contact time of the impact was 31 *μ*s, which is equal to one-half the time required for a P-wave to travel from the impact point to the epicenter. The waveform generated by a 31 *μ*s point impact is much smoother than the impulse response that was shown in figure 4. As the contact time increases, wave arrival times can become difficult to determine as displacements caused by individual waves become smeared together. As a result, sudden changes in the waveform will not necessarily correspond to the arrival times of the waves. (See refs. [3,4] for a more detailed discussion of the effect of contact time on surface displacement waveforms.)

The arrivals of P-, S-, and mode-converted wavefronts are indicated on the calculated waveform. The displacements caused by the large amplitude P-wave arrivals dominate the waveform. Notice that there is a second dip in the waveform after the end of the direct P-wave and before the arrival of the S-wavefront. The steady change in displacement (wake) between these two waves in the impulse response (see fig. 4) gives rise to this second dip.

#### Comparison With Finite Element Solution

The impact response of the same plate was also calculated using an axisymmetric finite element model. In both the Green’s function solution and the finite element analysis the plate was unsupported. Impact on the top surface of the plate was simulated by applying a uniform stress over an element at the center of the plate. The time history of the applied stress was a half-cycle sine curve with a duration of 31 *μ*s. The values of the material properties used in the analysis were: a modulus of elasticity of 33100 MPa, a Poisson’s ratio of 0.2, and a density of 2300 kg/m^3^. These values result in P-, S-, and R-wave speeds of 4000, 2440, and 2240 m/s, respectively. Figure 5(b) shows the normal displacement at the epicenter of the plate. The computed arrival times of P-, S-, and the mode-converted PS-wave are indicated on the waveform.

The response obtained by the finite element analysis can be compared with the Green’s function solution for an infinite plate for the period of time before wave reflections return from the sides of the bounded plate used in the finite element analysis. If the shape and magnitude of the perturbations in the waveform obtained from the Green’s function solution [fig. 5(a)] are compared with those obtained from the finite element analysis [fig. 5(b)], it is seen that there is good agreement between the two waveforms.

In the waveform obtained from the finite element analysis, there is a series of low amplitude, extraneous oscillations (ringing) between 128 *μ*s and the arrival of the 3P-wavefront. This ringing is due to the excitation of spurious modes of vibration in the constant strain finite elements used in DYNA2D. These modes are referred to as “zero energy” or “hourglass modes” [14] and they are due to distortions of the elements. A decrease in the contact time of the impact causes more rapid changes in displacement; this causes distortion of elements and tends to increase ringing. Artificial viscosities are introduced in DYNA2D to damp out the ringing [23], but it generally cannot be completely suppressed. The ringing is particularly evident in this case because there is a relatively quiescent period between the rapid change in displacements which occurs prior to 128 *μ*s and the arrival of the 3P-wave.

### Displacement Fields Within a Plate

A single finite element analysis solves for displacements and stresses over the entire domain (the collection of finite elements) at each time step during the specified time of analysis. These results can be used to study the dynamic displacement and stress fields that are produced within a solid.

An axisymmetric, finite element analysis was performed for a 25 *μ*s duration impact on a 0.5 m thick, 1.5 m diameter, unsupported, plate. Material properties were identical to those used in the previous analysis. A 0.5 m thick plate was used in this analysis so that the P- and S-waves generated by the 25 *μ*s contact time became separated as they propagated through the plate; this makes the displacement fields created by the waves easier to study.

**Figure 6:**
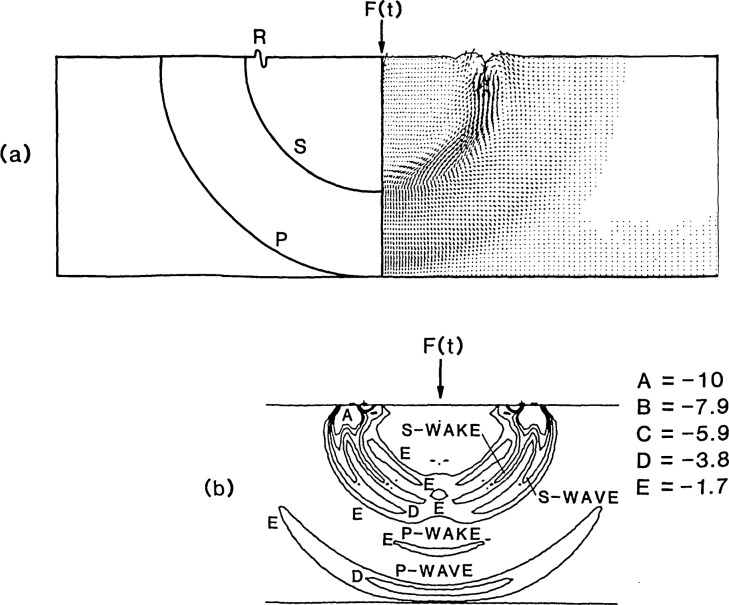
Displacement and stress fields within a 0.5 m thick plate 125 *μ*s after the start of the impact: (a) vector plot of displacements and the location of the waves; and (b) minimum principal stress contour plot.

**Figure 7:**
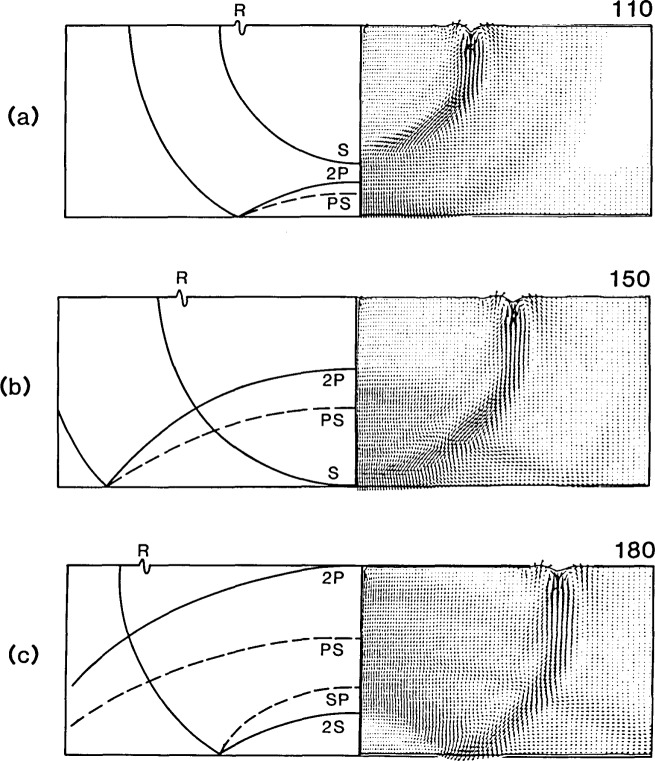
Vector plots of displacements at various times after the start of the impact: (a) 148 *μ*s; (b) 203 *μ*s; and (c) 250 *μ*s.

The righthand side of figure 6 shows the displacement field in the plate 125 *μ*s after the start of the impact. (Since the displacement field is axisymmetric, only half of the plate is shown.) At 125 *μ*s the P-wavefront arrives at the epicenter of the plate. The position of the P- and S-wavefronts are indicated on the lefthand side of the figure. The magnitude and direction of the average nodal dis placement of each element is indicated by a vector. The relative lengths of the vectors depend on the magnitude of the largest displacement that occurs within the plate at a particular time. The vector lengths are also adjusted by a scale factor which is not under the user’s control. Therefore, the vector plots shown in figures 6(a) and 7 are not drawn to the same scale; this must be remembered when comparing the figures.

As mentioned, motion in a P-wave is parallel to the direction of wave propagation. In figure 6, the vectors within the P-wave are oriented along rays emanating from the impact point. This orientation is consistent with the direction of motion. The magnitude of the displacements in the P-wave are not uniform along the spherical wave. Displacements are maximum near the centerline of the plate (the ray connecting the impact point to the epicenter) and they diminish to almost zero at the top surface of the plate. This pattern of displacements is in agreement with that shown in figure 2.

The motion in an S-wave is perpendicular to the direction of wave propagation. In figure 6, the S- wave is easy to identify because of the orientation and large amplitude of the vectors within the wave. As expected, vectors are perpendicular to rays emanating from the impact point. Displacements along a spherical surface within the plate were studied to determine the effects caused by the S-wave. The displacements in the S-wave are approximately zero at the center of the plate and become larger along rays located at increasing angles from the centerline. A study of displacement time- histories obtained for various elements along a spherical front inside the plate showed that, near the critical angle (approximately 37 degrees from the centerline of the plate), there is a discontinuity in the displacements caused by the arrival of the S-wave; this discontinuity agrees with that predicted by the radiation pattern shown in figure 2. Near the surface, it is difficult to determine the amplitude of the displacements in the S-wave because of interference due to the displacements caused by the R-wave. Note that in the vector displacement field all effects are superimposed; each vector represents the total displacement and direction of any given element.

Figure 6(b) shows a contour plot of minimum principal (compression) stress. The stresses in the P-wave are greatest at the centerline of the plate and decrease toward the surface. Since a state of pure shear stress is equivalent to a state of equal biaxial tension and compression, the plot of minimum principal stress also shows the stress variation in the S-wave. The stresses in the S-wave are lowest at the centerline and increase toward the surface. In the region near the surface of the plate, the stresses caused by the R-wave interfere with those produced by the S-wave making it difficult to separate the stresses caused by each wave.

The observed patterns of displacements and stresses in the P- and the S-waves are similar to those expected based on the displacement fields produced by a harmonic point source (fig. 2). However, in addition to these P- and S-wave radiation patterns, figure 6(b) shows that in the region between the P- and S-waves there are stresses that resemble those that occur in a P-wave; this is the “P-wake.” In addition, there is a region of nonzero displacements and stresses trailing the S-wave (the “S-wake”) that resembles the patterns in the S- wave. Thus, the disturbances generated by impact on a plate are not confined in the P- and S-waves.

Figures 7(a) through 7(c) show displacement fields obtained from the finite element analysis, along with corresponding schematic representations of the position of the P-, S-, and the mode- converted PS- and SP-waves, at 148, 203, and 250 *μ*s after the start of the impact.

The displacement field at 148 *μ*s [fig. 7(a)], shows reflection of the P-wave at the bottom surface of the plate. The S-wave created by mode- conversion of the incident P-wave (referred to as the PS-wave) is not yet discernible as it is masked by the displacements caused by the reflected P- wave.

At 203 *μ*s [fig. 7(b)], the S-wavefront arrives at the epicenter. The bottom surface of the plate is displaced downward at this time because of the effect of the preceding P-wave and P-wake. The S- wake is clearly visible.

At 250 *μ*s [fig. 7(c)], the front of the reflected P-wave arrives at the top surface of the plate. The PS-wave is now easily discernible. Reflection of the S-wave is occurring and the mode-converted P-wave (referred to as SP) that was generated by the reflection of the incident S-wave is seen emerging from the front of the reflected S-wave. The SP-wave causes much larger displacements than the reflected S-wave.

Once multiple reflections of the P-, S-, and mode-converted waves begin to occur, the disturbances created by individual waves become more difficult to distinguish in the displacement fields.

### Impact-Echo Response

The use of the impact-echo method for nondestructive testing involves interpretation of displacement waveforms obtained near the point of impact. In this section, a surface displacement waveform obtained from the Green’s function solution for the impact response of an infinite plate is compared with results obtained by the finite element method.

#### Green’s Function Solution

**Figure 8:**
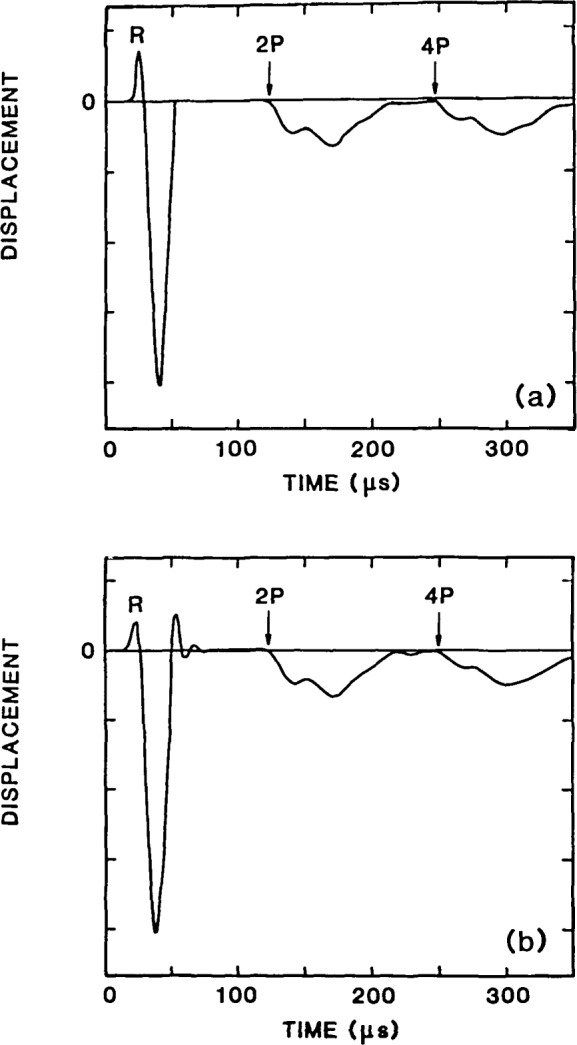
Impact-echo response: (a) Green’s function solution; and (b) waveform obtained for finite element analysis.

The normal displacement calculated at a point on the top surface of a infinite plate due to impact at another point on the same surface is shown in figure 8(a). This thickness, *T*, of the plate was 0.25 m. The spacing, *H*, between the impact point and the point where the displacement was calculated (the point where a receiving transducer would be located) was 0.05 m [see fig. 3(b)]. The ratio of the S- to the P-wave speed was 0.61 and the contact time of the impact was 31 *μ*s as in the epicenter analysis.

The waveform shown in figure 8(a) consists of displacements caused by the arrival of multiply reflected P- and S-waves and mode-converted waves. In addition, there is an initial large displacement caused by the R-wave propagating along the top surface of the plate. In the figure, the R-wave arrival is denoted by an R, and multiple P- and S-wave arrivals and mode-converted wave arrivals are indicated. For this particular configuration (*H/T*=0.2), the normal displacements caused by the S-wave are very small.

As discussed previously, the P-waves which arrive at the top surface are tension waves; the arrival of each tension wave pulls the surface downward. Thus the perturbations in the impactecho response have a pattern similar to the epicenter response.

#### Comparison With Finite Element Solution

Figure 8(b) shows the displacement waveform obtained from the finite element analysis of the 0.25 m thick plate subjected to a 31 *μ*s duration impact. The spacing between the impact and the point where the displacement waveform was recorded is 0.05 m as in the Green’s function solution. Material properties are the same as those used in the previous analyses.

If the shape and relative magnitudes of the perturbations in the waveform obtained from the finite element analysis [fig. 8(a)] are compared with those in the Green’s function solution [fig. 8(b)], it is seen that there is good agreement between the two waveforms.

As in the epicenter response obtained from the finite element analysis, low amplitude oscillations due to excitation of the zero energy modes of the finite elements occur in figure 8(b). After the R- wave has passed the receiver, the surface displacement should go to zero, as shown in figure 8(a), until reflections arrive from the bottom of the plate. However, the oscillations due to excitation of the zero energy modes cause the computed surface displacement to oscillate about zero for a short time. In this case, the zero energy modes are excited by the element distortion caused by the rapid, large changes in displacement that occur in the R-wave. This numerical ringing does not affect the echo pattern due to the multiply reflected waves.

## Summary

The internal displacement and stress fields produced by a transient point load on the top surface of an elastic plate were studied using the finite element method. It was shown that in addition to P- and S-waves, intermediate displacement and stress fields (wakes) are produced by a transient point load applied normal to a stress-free boundary. Surface displacement waveforms computed by the finite element method showed good agreement with those obtained from the Green’s function solution.

The study presented in this paper has demonstrated the potential of the finite element method for becoming a powerful tool for understanding the interaction of stress waves with defects within solids. Such knowledge is essential for successful implementation of nondestructive testing techniques based on stress wave propagation, such as the impact-echo method under development at NBS. The power of the finite element method lies in its ability to analyze solids having arbitrary shapes, boundary conditions, and applied loads, and to generate complete pictures of displacement and stress fields in a computationally efficient manner. A subsequent paper will present finite element studies of the diffraction of transient stress waves by flat-bottom holes and circular disks within plates—problems for which no Green’s function solutions currently exist.

## Footnotes

1 Figures in brackets indicate literature references.

2 Generalized Ray Theory was originally developed in the 1960’s for geophysical applications, but is readily applicable to the study of transient waves in bounded solids. For an introduction to the ideas and formulation of ray theory, see ref. [9].

## References

[1] Carino NJ, Sansalone M (1984). Pulse-Echo Method for Flaw Detection in Concrete.

[2] Carino NJ, Malhotra VM (1984). Laboratory study of flaw detection in concrete by the pulse-echo method. InSitu/Nondestructive Testing of Concrete.

[3] Carino NJ, Sansalone M, Hsu NN (1986). A point source-point receiver, pulse-echo technique for flaw detection in concrete. Journal of the American Concrete Institute.

[4] Carino NJ, Sansalone M, Hsu NN, McGonnagle W (1986). Flaw detection in concrete by frequency spectrum analysis of impact-echo waveforms. International Advances in Nondestructive Testing.

[5] Kolsky H (1963). Stress Waves in Solids.

[6] Roderick RL (1951). The radiation pattern from a rotationally symmetric stress source on a semi-infinite solid. Ph D Thesis, Grad Div of Appl Math.

[7] Miller GF, Pursey H (1954). The field and radiation impedance of mechanical radiators on the free surface of a semi-infinite solid. Proc of the Royal Society of London, A.

[8] Miller GF, Pursey H (1955). On the partition of energy between elastic waves in a semi-infinite solid. Proc of the Royal Society of London, A.

[9] Pao YH, Gajewski RR, Mason WP, Thurston RN (1977). The generalized ray theory and transient responses of layered elastic solids. Physical Acoustics.

[10] Johnson LJ (1974). Green’s function for Lamb’s problem. Geophysical J Royal Astro Soc.

[11] Pao YH, Gajewski RR, Ceranogly AN (1979). Acoustic emission and transient waves in an elastic plate. J Acoust Soc Am.

[12] Gallagher RH (1975). Finite Element Analysis, Fundamentals.

[13] Hallquist JO (1976). A Procedure for the Solution of Finite-Deformation Contact-Impact Problems by the Finite Element Method, UCRL-52066.

[14] Goudreau GL, Hallquist JO (1982). Recent developments in large-scale finite element Lagrangian hydrocode technology, Computer Methods in App. Mechanics and Eng.

[15] Hallquist JO (1984). User’s Manual for DYNA2D—An Explicit Two Dimensional Hydrodynamic Finite Element Code With Interactive Rezoning.

[16] Hallquist JO (1983). User’s Manual for MAZE: An Input Generator for DYNA2D and NIKE2D.

[17] Hallquist JO (1983). User’s Manual for ORION: An Interactive Post-Processor for the Analysis Codes NIKE2D, DYNA2D, and TAC02D.

[18] Goldsmith W (1965). Impact: The Theory and Practice of Colliding Solids.

[19] Bracewell RN (1978). The Fourier Transform and Its Application.

[20] Eitzen D (1982). Fundamental Developments for Quantitative Acoustic Emission Measurements.

[21] Hsu NN (1985). Dynamic Green’s Function of an Infinite Plate—A Computer Program.

[22] Graff K (1975). Wave Motion in Elastic Solids.

[23] Flannagan DP, Belytschko T (1981). A uniform strain hexahedron and quadrilateral with orthogonal hourglass control. Int Journal of Numerical Methods in Engineering.

